# Learning to read Chinese promotes two cortico-subcortical pathways: The development of thalamo-occipital and fronto-striatal circuits

**DOI:** 10.3389/fnins.2022.983084

**Published:** 2022-08-24

**Authors:** Yanpei Wang, Jie Luo, Leilei Ma, Rui Chen, Jiali Wang, Congying Chu, Weiwei Men, Shuping Tan, Jia-Hong Gao, Shaozheng Qin, Yong He, Qi Dong, Sha Tao

**Affiliations:** ^1^State Key Laboratory of Cognitive Neuroscience and Learning, Beijing Normal University, Beijing, China; ^2^IDG/McGovern Institute for Brain Research, Beijing Normal University, Beijing, China; ^3^Department of Psychological Sciences, University of Connecticut, Storrs, CT, United States; ^4^Center for MRI Research, Academy for Advanced Interdisciplinary Studies, Peking University, Beijing, China; ^5^Psychiatry Research Center, Beijing HuiLongGuan Hospital, Peking University, Beijing, China

**Keywords:** learning to read, thalamo-occipital circuits, fronto-striatal circuits, school-age children, longitudinal development

## Abstract

Learning to read may result in network reorganization in the developing brain. The thalamus and striatum are two important subcortical structures involved in learning to read. It remains unclear whether the thalamus and striatum may form two independent cortico-subcortical reading pathways during reading acquisition. In this prospective longitudinal study, we aimed to identify whether there may be two independent cortico-subcortical reading pathways involving the thalamus and striatum and to examine the longitudinal predictions between these two cortico-subcortical pathways and reading development in school-age children using cross-lagged panel modeling. A total of 334 children aged 6–12 years completed two reading assessments and resting functional imaging scans at approximately 12-month intervals. The results showed that there were two independent cortico-subcortical pathways, the thalamo-occipital and fronto-striatal circuits. The former may be part of a visual pathway and was predicted longitudinally by reading ability, and the prediction was stronger in children in lower grades and weaker in children in higher grades. The latter may be part of a cognitive pathway related to attention, memory, and reasoning, which was bidirectionally predicted with reading ability, and the predictive effect gradually increasing with reading development. These results extend previous findings on the relationship between functional connectivity and reading competence in children, highlighting the dynamic relationships between the thalamo-occipital and fronto-striatal circuits and reading acquisition.

## Introduction

Learning to read may result in brain network reorganization (Houde et al., 2010; Martin et al., 2015). In one resting-state functional magnetic resonance imaging (fMRI) study of illiterate adults, Skeide et al. (2017) observed that a 6-month literacy intervention altered the cortico-subcortical crosstalk in the visual system of illiterate individuals. Some studies, including studies based on comparisons between children and adults (Koyama et al., 2020), literacy training studies (Alcauter et al., 2017; Hancock et al., 2017; Koyama et al., 2020; Mohammadi et al., 2020), and child development studies, (Alcauter et al., 2017) also found that cortico-subcortical alterations play an important role at the early stage of learning to read. Among these alterations, the most frequently affected subcortical structures were the striatum and thalamus. However, no study has explored the respective roles of cortico-thalamic and the cortico-striatal connection in the early stages of reading development.

The thalamus is a large mass of gray matter located in the dorsal part of the diencephalon. Nerve fibers project out of the thalamus to the cerebral cortex in various directions, allowing hub-like exchanges of information. The thalamus is critical for the detection of visual changes (Rima and Schmid, 2020). Effective temporal and spatial interpretation of text by the top-down attention network of the visual system is a critical early stage of reading, and any lesions that impair this process can lead to dyslexia (Vidyasagar, 2019). Actually, there is evidence that the important role of the thalamus is mainly reflected early in the stage of reading development. Koyama et al. (2011) found that the thalamus is specific to reading processing brain regions in children based on the results of two meta-analyses (Bolger et al., 2005; Houde et al., 2010). Siok et al. (2020) depicted a lifespan developmental trajectory of the activation intensity of related brain regions during reading task execution, which indicated that thalamic activation is gradually reduced. Correlation analysis of the thalamo-cortical visual pathway (functional connectivity) and reading competence showed a significant positive relationship in children but a non-significant negative association in adults (Koyama et al., 2011). This suggests that children’s reading ability relies on the thalamo-cortical visual pathway, which does not appear to be necessarily beneficial for reading in adults. However, how the role of the thalamo-cortical visual pathway in learning to read is gradually changing and the bidirectional relationship between the two regions remains unclear, which requires further confirmation from a longitudinal study.

The striatum, as a part of the basal ganglia, receives information from the cortex and forms the corticostriatal loop projecting to the frontal lobe. The striatum is associated with semantic, phonological, and articulatory processing while reading (Xu et al., 2005; Binder et al., 2006; Bitan et al., 2007; Brem et al., 2009). The dysfunction of fronto-striatal circuits was confirmed by a meta-analysis to cause fundamental impairments in reading-related processing (Hancock et al., 2017). Fronto-striatal functional connectivity was significantly weaker in illiterate individuals than in literate controls (Mohammadi et al., 2020). Some previous studies have suggested that the striatum may be more involved in reading in adults than in children. For example, a lifespan fMRI study found that the striatum was activated in adults when reading but not in children (Siok et al., 2020). Several meta-analyses of reading task-based fMRI studies failed to identify striatum activation in children (Houde et al., 2010; Li and Bi, 2022) and found striatum activation specific to adults (Richlan et al., 2011). However, a study found that fronto-striatal functional connectivity significantly predicted reading performance in children aged 6-9 years (Alcauter et al., 2017). Thus, the striatum may be mainly activated for adult reading, and the fronto-striatal circuits may also be involved in reading when children are learning to read. However, the above studies are based on cross-sectional data, and how the roles of the fronto-striatal circuit develop and change during when learning to read remains unclear and requires longitudinal studies to offer clear evidence.

The thalamo-occipital circuit is an important visuospatial pathway and involves visual processing and visual pathway reorganization in early reading (Muller-Axt et al., 2017; Skeide et al., 2017; Tschentscher et al., 2019), and its damage can cause blindsight and developmental dyslexia (Rima and Schmid, 2020). Fronto-striatal connectivity is a critical cortico-subcortical pathway involved in language and cognitive processing (Gordon et al., 2021). The fronto-striatal pathway has been shown to be closely associated with a variety of cognitive abilities, including inhibitory control (Ojha et al., 2022), working memory(Rodrigue et al., 2020; Hidalgo-Lopez and Pletzer, 2021), executive function (Galandra et al., 2019), and cognitive flexibility (Banaie Boroujeni, 2021); its impairment can lead to problems, such as attention-deficit/hyperactivity (Cupertino et al., 2020; Mamiya et al., 2021) and reading disorder (Hancock et al., 2017). From recent evidence, the thalamo-occipital and fronto-striatal connectivities seem to be two functional independent pathways. However, it is not clear whether the two pathways maintain functional independence in the processes of learning to read and further developing reading skills.

Based on the previous research mentioned above, there may be two important cortico-subcortical pathways relevant to learning to read that may involve the thalamus and striatum. More research is needed to examine how thalamo-cortical and cortico-striatal pathways play roles in children’s process of learning to read. In this study, we conducted a longitudinal brain-behavioral study among several hundred school-aged children, varying in ability from beginning readers to intermediate readers, and used cross-lagged panel analyses to explore how the two cortico-subcortical pathways and reading development may predict each other over one year. Further association analyses were conducted to examine the cognitive basis of both pathways. We hypothesized that the thalamo-occipital and fronto-striatal circuits may be two important pathways involved in learning to read. The former may be part of visual-spatial processing that plays important roles in the early stage of learning to read and gradually weakens with the development of reading ability. In contrast, the latter may be a complex cognitive pathway that always plays an important role in learning to read and gradually strengthens with the development of reading.

## Materials and methods

### Participants

Neuroimaging and behavioral data were obtained from the Children School Functions and Brain Development Project (CBD, Beijing Cohort: Tao, 2019). Comprehensive assessments have been conducted yearly, including MRI brain scans, reading achievement, cognition and others. Children were recruited from dozens of primary schools in Beijing. Informed consent was obtained from the parents or guardians (written) and children (oral). The exclusion criteria included a history of neurological or psychiatric disorders, the use of psychoactive drugs, significant head injury, and physical illness that prevented MRI scanning. All study procedures were reviewed and approved by the Institutional Review Boards at Beijing Normal University in accordance with the Declaration of Helsinki.

This study included 334 children with complete MRI scans and reading and cognition scores at both baseline and one-year follow-up assessments. More detailed information about the participants is presented in Table 1. Referring to a previous study (Siok et al., 2020), we categorized the participants as beginning readers (grade 3 and below, *n* = 167) and intermediate readers (grade 4 and above, *n* = 167).

**TABLE 1:** Characteristics of the participants at baseline and follow-up

	Baseline (*n* = 334)	Follow-up (*n* = 334)	*t* value
Age (mean ± SD)	9.03 ± 1.33	10.20 ± 1.41	
Sex:Females, *n* (%)	157(47.0%)		
Parental Education (mean ± SD)	8.82 ± 2.55		
Family Income (mean ± SD)	8.80 ± 2.72		
Reading achievements (mean ± SD)	524.34 ± 98.27	548.45 ± 105.43	4.37***
Attention (mean ± SD)	93.59 ± 10.64	100.90 ± 11.38	13.87***
Memory (mean ± SD)	91.42 ± 11.07	97.71 ± 13.59	9.18***
Visuospatial Perceptive (mean ± SD)	97.97 ± 12.83	103.90 ± 13.06	9.52***
Reasoning (mean ± SD)	95.99 ± 11.99	100.09 ± 12.44	7.03***

Follow-up = the assessment after one year. The parent’s education level refers to the highest level of education between children’s parents. Parental Education: 1 = Uneducated; 2 = Primary education; 3 = Junior school; 4 = High school; 5 = Secondary vocational school; 6 = Polytechnic school; 7 = Higher vocational education; 8 = Junior college(part-time); 9 = Junior college(full-time); 10 = Bachelor degree (part-time); 11 = Bachelor degree (full-time); 12 = Graduate education or above. Family Income (RMB/year): 1 = Less than 3,000; 2 = 3,001–6,000; 3 = 6,001–10,000; 4 = 10,001–30,000; 5 = 30,001–50,000; 6 = 50,001–100,000; 7 = 100,001–150,000; 8 = 150,001–200,000; 9 = 200,001–400,000; 10 = 400,001–600,000; 11 = Over 600,000. *p < 0.05, **p < 0.01, ***p < 0.001.

### Reading achievement test

Based on the national curriculum, the reading achievement test was developed by the project team of the National Children’s Study of China (NCSC) (Dong and Lin, 2011). It assessed character and word recognition as well as sentence and short passage comprehension. Item response theory (IRT) scores, with an average of 500 and a standard deviation of 100, were computed based on the comprehensive national representative sample of 140,000 children and adolescents in more than 600 primary and junior high schools from 100 counties and 31 provinces around mainland China. According to the technical report of the NCSC (Dong and Lin, 2011) as well as previous research (Wang et al., 2022), this test showed good psychometric properties. The Cronbach’s alpha coefficients were 0.72–0.94 at various grades, and the average difficulty coefficient was 0.69. Children completed the test in small groups on a computer within 45 min.

### Cognitive abilities

The cognitive assessment battery developed by the NCSC project team (Dong and Lin, 2011) was used. There are four subtests, including attention, memory, visuospatial perception, and reasoning. This battery has been used in previous studies (Ren et al., 2013, 2015; Tao et al., 2015; Wang et al., 2016).

#### Attention

This subtest consists of four sets of number cancellations. In each set, participants were asked to cross out a number with specific marks from 200 items randomly arranged and presented within a 20 × 10 matrix that included 44 targets among the non-targets within 1 min. The correlation between this test and the Cancellation subtest of the Wechsler Intelligence Scale for Children (WISC-IV, Chinese version) (Zhang, 2009) was 0.72 (*p* < 0.01) among 114 children (Dong and Lin, 2011). The raw score was computed by subtracting the number of false hits from the total number of hits and transferred into a norm-based standardized score based on the national representative datasets. The internal consistency (Cronbach’s α) was 0.94.

### Memory

This subtest consists of 27 items, among which 12 are number recognition, and 15 are object pair recognition. Participants were asked to select numbers or matched objects presented previously from alternatives immediately and with a delay of 30 min, respectively. Among 110 children, the correlations between this memory test and the memory subtest of the WISC-IV (WISC-IV, Chinese version) (Zhang, 2009) were 0.53 (*p* < 0.01) and 0.46 (*p* < 0.01) for the number and object tests, respectively (Dong and Lin, 2011). The total number of correct responses was transferred into a norm-based standardized score based on the national representative datasets. The internal consistency (Cronbach’s α) was 0.81 for the number recognition test and 0.74 for the object pair test.

#### Visuospatial perception

There were 27 items, among which 11 are hidden figures and 16 are mental rotation. In the hidden figure subtest, participants were asked to assess 4 options and identify the figure that was not in the complex figure presented previously. In the mental rotation subtest, participants were asked to identify the rotated figure among the 4 options that had been presented previously. Among 116 children, the correlation between the outcomes of the hidden figure subtest and the Test of Visual Perceptual Skills (TVPS-3) was 0.51 (*p* < 0.01), and the correlation between the outcomes of the mental rotation subtest and the Motor-free Visual Perceptual Test (MVPT-3) was 0.57 (*p* < 0.01) (Dong and Lin, 2011). The number of correct responses was transferred into a norm-based standardized score based on the national representative datasets. The internal consistency (Cronbach’s α) was 0.74 for the hidden figure subtest and 0.77 for the mental rotation subtest.

#### Reasoning

This subtest consists of 40 items of figures and numbers. Participants were asked to choose one of four alternatives to complete figure or number sequences according to the rules embedded in the presented figure or number sequences. Among 111 children, the correlations among the figural reasoning subtest, the numerical reasoning subtest and the matrix reasoning subtest of the WISC-IV (WISC-IV, Chinese version) (Zhang, 2009) were 0.66 (*p* < 0.01) and 0.64 (*p* < 0.01), respectively, (Dong and Lin, 2011). The number of correct responses was transferred into norm-based standardized scores based on the national representative datasets. The internal consistency (Cronbach’s α) was 0.77 for the figural reasoning test and 0.86 for the numerical reasoning test.

### Image acquisition

All MRI scans were acquired on two 3T Siemens Prisma scanners with a 64-channel head coil at Peking University and Beijing HuiLongGuan Hospital using the same imaging sequences. Blood oxygen level-dependent (BOLD) fMRI data were acquired using a whole-brain, single-shot, multislice, echo-planar imaging (EPI) sequence of 240 volumes with the following parameters: repetition time/echo time (TR/TE) = 2000/30 ms, flip angle = 90°, field of view (FOV) = 224 × 224 mm, matrix = 64 × 64, slice thickness = 3.5 mm, and slices = 33. The resulting nominal voxel size was 3.5 mm × 3.5 mm × 3.5 mm. A fixation cross was displayed as images were acquired. Subjects were instructed to remain awake, keep their eyes open, fixate on the displayed blank screen, and remain still. Prior to time-series acquisition, a 6-min magnetization-prepared, rapid acquisition gradient-echo T1-weighted (MPRAGE) image (TR = 2530 ms, TE 2.98 ms, FOV 256 mm × 224 mm, matrix, effective voxel resolution of 1 mm × 1 mm × 1 mm, slice thickness = 1 mm, and slices = 192) was acquired to aid spatial normalization to standard atlas space. Prior to scanning, to acclimate subjects (children) to the MRI environment, a mock scanning session was conducted for each individual using a decommissioned MRI scanner and head coil. Mock scanning was accompanied by acoustic recordings of the noise produced by gradient coils for each scanning pulse sequence. To further minimize motion, subjects’ heads were stabilized in the head coil using one foam pad over each ear.

### MRI quality control

All MRI scan quality control procedures are described below. (i) Individual images were subjected to a careful visual examination performed by an experienced radiologist to exclude incidental abnormalities, such as arachnoid cysts, neuroepithelial cysts and other intracranial space-occupying lesions. (ii) Careful visual inspections with a scan rating procedure were separately conducted by five experienced raters using a protocol similar to that used in the Human Connectome Project (Marcus et al., 2013). (iii) Images considered to have a better than fair quality by both raters were retained. We quantified the head motion during resting-state fMRI acquisition as framewise displacement (FD) (Power et al., 2012). The participants were also excluded if the mean FD exceeded 0.5 mm during resting-state scans (Xia et al., 2018). In this study, a total of 12 children have been excluded from the data analysis because of substandard quality control.

### Image data analysis

Resting fMRI preprocessing was performed using DPARSF software^1^ (Yan and Zang, 2010). Preprocessing included the following steps: (1) slice-timing correction; (2) head-motion correction; (3) spatial normalization (MNI); (4) whole-brain and white matter signals and 24 motion parameters being regressed out; (5) spatial smoothing with a 6-mm 3D full-width half-maximum kernel; and (6) temporal bandpass filtering (0.01–0.1 Hz).

Literature-based spherical seed regions with a radius of 4 mm were created using DPARSF (Yan and Zang, 2010) in the bilateral thalamus [Montreal Neurological Institute (MNI) coordinates, left: x = 6, y = −18, z = −3; right: x = −6, y = −21, z = −3] (Skeide et al., 2017) and the bilateral striatum (MNI coordinates, left: x = −18, y = 18, z = 0; right: x = −10, y = 14, z = 8) (Alcauter et al., 2017). Mean time series were extracted by averaging the time series of all voxels in the seed region, and the correlation coefficients between this time course and all other brain voxels were computed. The correlation maps were then z-normalized using Fisher’s *r*-to-*z* transformation to approximate a normal distribution. In addition, the Automated Anatomical Labeling (AAL) atlas was used for anatomical labeling of the MRI peaks/clusters in this study.

### Statistical analysis

Cross-lagged panel analyses were performed using AMOS 21.0 (IBM). All statistical analyses of MRI data were performed in DPABI software (see footnote 1) (Yan et al., 2016). Pearson’s correlation was used to evaluate seed-based connectivity, with a significance threshold set at a voxel-size value of *p* < 0.001 and a familywise error-corrected cluster probability of P < 0.05. Brain-behavior correlations were performed using SPSS 21 (IBM) with a significance threshold set 0.0125 (Bonferroni correction: alpha/number of tests = 0.05/4 = 0.0125). The directional association between functional connectivity and reading ability was determined by the cross-lagged panel model (CLPM) (Hamaker et al., 2015). Age, sex, handedness, site, household income, parental education, and head motion were controlled as covariates.

## Results

### Sample characteristics

All sample characteristics are presented in Table 1. Both reading (*t* = 4.37, *p* < 0.001) and cognitive abilities (attention, memory visuospatial perception and reasoning, all *t*s ≥ 7.03, all *p*s < 0.001) performance increased at the follow-up assessment compared with that at the baseline assessment (Table 1).

### The two cortico-subcortical reading pathways were identified: Thalamo-occipital and fronto-striatal circuits

**FIGURE 1:**
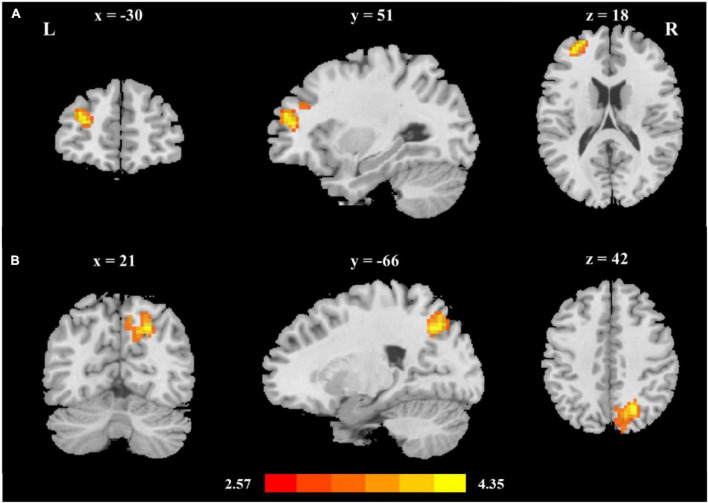
The two cortico-subcortical reading pathways **(A)** Reading ability was related to fronto-striatal functional connectivity (left middle frontal gyrus, MNI coordinates: x = −30, y = 51, z = 18); **(B)** Reading ability was related to thalamo-occipital functional connectivity (right superior occipital gyrus, MNI coordinates: x = 21, y = −66, z = 42). Age, sex, handedness, site, household income, parental education, and head motion were used as covariates of no interest.

To explore the thalamus-occipital and fronto-striatal circuits in reading development, we performed seed-based functional connectivity analysis. First, the mean time series were extracted by averaging the time series of all voxels in the seed region, including the bilateral thalamus and striatum, and the correlation coefficients between this time course and all other brain voxels were computed. Then, brain-behavior correlations were performed between the *z value* maps and reading ability. Striatum-based analysis showed that reading ability was related to the functional connectivity between the left striatum and left middle frontal gyrus (MNI coordinates: x = −30, y = 51, z = 18; Figure 1A and Table 2); thalamus-based analysis revealed that reading was associated with the functional connectivity between the right thalamus and left superior occipital gyrus (MNI coordinates: x = 21, y = −66, z = 42; Figure 1B and Table 2). The right striatum and left thalamus-based analyses did not reveal significant results after correction for multiple comparisons.

**TABLE 2:** Seed-based functional connectivity related to reading.

Seed	Regions	HS	MNI Coordinate			Voxel	*z*-value
			x	y	Z		
Left striatum	MFG/SFG	L	−30	51	18	78	4.35
Right thalamus	PCUN/SOG	R	21	−66	42	244	4.18

MFG, middle frontal gyrus; SFG, superior frontal gyrus; PCUN, precuneus; SOG, superior occipital gyrus; L, left; R, right; HS, hemisphere.

### Longitudinal prediction between reading and the two cortico-subcortical pathways: CLPM analysis

**FIGURE 2:**
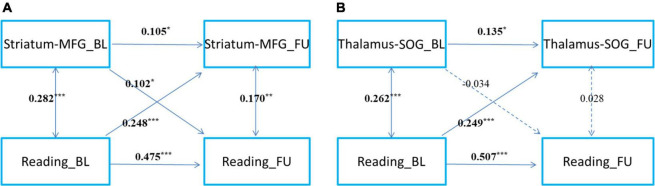
The cross-time predictions between cortical-subcortical functional connectivity and reading development in school-age children The cross-lagged panel models of **(A)** left striatum and left middle frontal gyrus connectivity, **(B)** right thalamus and superior occipital gyrus and reading development. MFG, middle frontal gyrus; SOG, superior occipital gyrus. Standardized estimates are presented. Age, sex, handedness, site, household income, parental education, and head motion were used as covariates of no interest. *p < 0.05, **p < 0.01, ***p < 0.001.

Children’s reading performance and two cortico-subcortical pathways (striatum-MFG and thalamus-SOG) at baseline and follow-up were significantly correlated (Table 3). CLPM analysis showed that reading and functional connectivity between the left striatum and left middle frontal gyrus could bidirectionally predict each other’s development one year later (Figure 2A), while reading could only unidirectionally predict the development of functional connectivity between the right thalamus and right superior occipital gyrus one year later (Figure 2B).

**TABLE 3:** The correlation matrix between reading and cortico-subcortical functional connectivity at baseline and follow-up assessments

		Striatum-MFG		Thalamus-SOG	
		BL	FU	BL	FU
Reading	BL	0.231***	0.210***	0.212**	0.191*
	FU	0.188***	0.198***	0.117*	0.161**

BL, baseline; FU, follow-up; MFG, middle frontal gyrus; SOG,superior occipital gyrus. Age, sex, handedness, site, household income, parental education, and head motion were used as covariates of no interest. *p < 0.05, **p < 0.01, ***p < 0.001.

**FIGURE 3:**
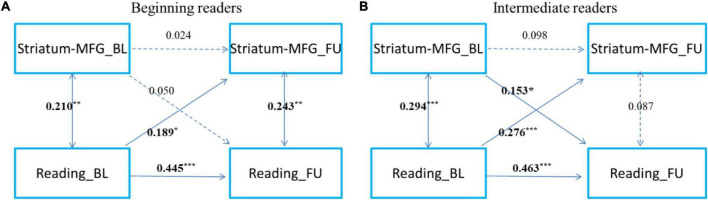
The cross-time predictions between left striatum and left middle frontal gyrus functional connectivity and reading development in school-age children The cross-lagged panel models in **(A)** beginning readers (grade 3 and below), and **(B)** intermediate readers (grade 4 and above). Standardized estimates are presented. MFG, middle frontal gyrus. Age, sex, handedness, site, household income, parental education, and head motion were used as covariates of no interest. *p < 0.05, **p < 0.01, ***p < 0.001.

**FIGURE 4:**
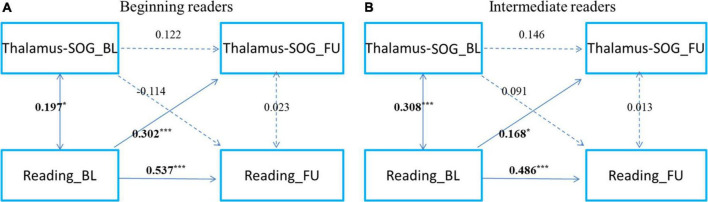
The cross-time predictions between right thalamus and right superior occipital gyrus functional connectivity and reading development in school-age children The cross-lagged panel models in **(A)** beginning readers (grade 3 and below), and **(B)** intermediate readers (grade 4 and above). Standardized estimates are presented. SOG, superior occipital gyrus. Age, sex, handedness, site, household income, parental education, and head motion were used as covariates of no interest. *p < 0.05, **p < 0.01, ***p < 0.001.

To examine the development of and change in the relationship, we categorized readers into primary reading and intermediate reading groups. To further examine the development and change in the relationship between cortical-subcortical functional connectivity and reading, referring to a previous study (Siok et al., 2020), we categorized the participants as beginning readers (grade 3 and below, *n* = 167) and intermediate readers (grade 4 and above, *n* = 167) (Figures 3, 4). First, we found that reading could predict the connectivity between the left striatum and left middle frontal gyrus both in beginning readers and intermediate readers, and the effect did not show significant differences between the two groups (χ*^2^* = 0.375, *p* = 0.540), while the reverse prediction was only significant in intermediate readers (Figure 3). Second, we found that reading could predict the connectivity between the right thalamus and right superior occipital gyrus both in beginning readers and intermediate readers, and the effect was weaker in intermediate readers (χ*^2^* = 5.447, *p* = 0.020), while the reverse prediction was not significant in either group (Figure 4).

### The cognitive basis of the thalamo-occipital and fronto-striatal circuits

**FIGURE 5:**
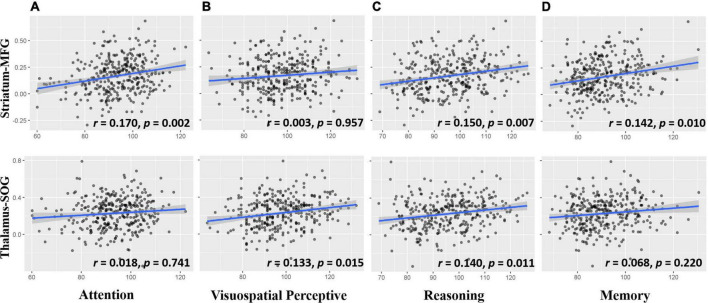
Scatter plots for the correlation between cortico-subcortical functional connectivity and cognitive ability **(A)** Attention ability related to striatum-MFG, but not thalamus-SOG; **(B)** visuospatial perceptive ability related to thalamus-SOG, but not striatum-MFG; **(C)** reasoning ability was associated with striatum-MFG and thalamus-SOG; **(D)** memory ability was associated with striatum-MFG, but not thalamus-SOG. The upper row is the striatum-MFG and the lower row is the thalamus-SOG. Striatum-MFG, functional connectivity between left striatum and left middle frontal gyrus; Thalamus-SOG, functional connectivity between right thalamus and right superior occipital gyrus. Age, sex, handedness, site, household income, parental education, and head motion were used as covariates of no interest.

To explore the cognitive basis of cortical-subcortical functional connectivity, we performed a partial correlation analysis controlling for age, sex, handedness, site, household income, parental education, and head motion. This analysis showed that the striatal frontal pathway was associated with attention, reasoning, and memory but not visuospatial perceptive ability (Figure 5 upper layer graph) and that the thalamic occipital pathway was related to visuospatial perceptive and reasoning but not attention and memory ability (Figure 5 lower layer graph).

Because attention, reasoning and memory tests contain visuospatial perceptive processing, to exclude its influence on the other three cognitive abilities, we controlled for visuospatial perceptive processing with other covariates and found that the correlations between the striatal frontal pathway and attention (*r* = 0.162, *p* = 0.003), between the striatal frontal pathway and reasoning (*r* = 0.163, *p* = 0.003), and between the striatal frontal pathway and memory (*r* = 0.149, *p* = 0.007) were still significant. In addition, the correlation between the thalamic occipital pathway and reasoning was no longer significant (*r* = 0.095, *p* = 0.086). This finding suggests that the relationship between the thalamic occipital pathway and reasoning observed in the reasoning test may be caused by visuospatial processing rather than reasoning processing.

## Discussion

This longitudinal study of reading development in school-age children identified two important cortico-subcortical pathways—thalamo-occipital and fronto-striatal circuits—and provided novel evidence for understanding the developmental connections between reading and cortico-subcortical crosstalk. We demonstrated that the thalamo-occipital and fronto-striatal circuits are two important pathways of learning to read. The former is a visual pathway that can be predicted by reading ability, and the prediction gradually weakens with the improvement in reading ability, while the latter is a complex cognitive pathway related to attention, memory, and reasoning. And it can predict each other with reading, and its predictive effect on reading increases with the improvement of reading ability.

### The fronto-striatal circuit: A cognitive pathway

The first pathway we found was the fronto-striatal cognitive pathway, which is formed by the functional connectivity between the left striatum and left middle frontal gyrus. This pathway has been thought to play an important role in reading, and a meta-analysis found that fronto-striatal abnormalities in reading disorders could arise from fundamental impairments in reading-related processes, such as phonological processing and implicit sequence learning, relevant to early language acquisition (Hancock et al., 2017). Consistent with our study, one cross-sectional, small sample study (*n* = 60) found that resting-state functional connectivity of the striatum and prefrontal cortex predicts reading performance in children aged 6–9 years old (Alcauter et al., 2017). However, in Alcauter et al.’s (2017) study, the prediction was not directional, simply providing a correlation based on cross-sectional data. In this study, using longitudinal cross-lagged panel analyses, we found that fronto-striatal circuitry and reading predicted each other in children aged 6–12 years. In addition, we found that fronto-striatal functional connectivity was predicted by learning to read at an early age and gradually became one of the important predictors of reading ability as one’s reading ability developed. We confirmed and advanced the conclusions of Alcauter et al. (2017). We found that the predictive relationship demonstrated in Alcauter et al.’s (2017) study was for the prediction of reading based on the fronto-striatal pathway, and the predictive effect of the fronto-striatal pathway on reading was not significant until after grade 3.

The left middle frontal gyrus was recognized as critical for Chinese reading and reading acquisition. Learning to read could increase the activation in the left middle frontal gyrus (Li et al., 2006; Siok et al., 2020), and activation and gray matter volume in this region decreased in dyslexic Chinese children (Siok et al., 2004, 2008, 2009, 2020; Tan et al., 2005; Xu et al., 2015). In this study, the fronto-striatal circuit was composed of the connection between the left middle frontal gyrus and the striatum. Is the fronto-striatal functional connectivity found in this study a pathway specific to Chinese reading? To answer this question, we need to address three related issues. First, the middle frontal gyrus found in this study is not the middle frontal gyrus specific for Chinese reading; the Chinese reading-specific brain region found by Tan et al. (2005) is located in BA9, while this study found that the region is located in BA46. Second, does this pathway only exist in Chinese reading and not in alphabetic languages? In fact, the answer is no. Several studies based on alphabetic language reading have found that this pathway plays an important role (Alcauter et al., 2017; Hancock et al., 2017; Mohammadi et al., 2020). Third, is fronto-striatal connectivity stronger in Chinese reading than in alphabetic language reading? This study was unable to answer this question due to the lack of data on reading alphabetic languages. This question may need to be answered in future bilingual studies. In conclusion, recent evidence does not support fronto-striatal connectivity as a pathway specific to Chinese reading, and further research is needed to explore whether this pathway is more important in Chinese reading than in alphabetic languages.

### The thalamo-occipital circuit: A visual pathway

The second pathway we found was the thalamo-occipital visual pathway formed by the functional connectivity between the right thalamus and right superior occipital gyrus. This was consistent with a previous study that trained illiterate individuals to be literate and found that training improved the degree of the thalamic activity and the strength of its connection to the occipital lobe (Skeide et al., 2017). In addition, we further investigated the predictive relationship between reading ability and the thalamo-occipital pathway and found that the predictive effect of reading on the thalamo-occipital pathway was unidirectional in both beginning and intermediate readers, and the predictive effect decreased gradually with reading ability. This finding suggests that the thalamo-occipital pathway may be closely related to visual processing in early reading. Effective temporal and spatial interpretation of text by the visual system is a critical early stage of reading, and any lesions that impair this process can lead to dyslexia, including downstream effects on the phonological domain (Vidyasagar, 2019). In this study, we only found that reading promoted the thalamo-occipital pathway but did not find the thalamo-occipital pathway to support or restrict the development of reading ability. This may be mainly because our samples were all developing children whose visual system and reading ability were well developed; therefore, there was no significant predictive effect on reading.

In this study, we found two cortico-subcortical pathways, thalamo-occipital and fronto-striatal circuits; however, in the study of literacy training, only the thalamus and thalamo-occipital pathways were observed (Skeide et al., 2017). Why did the authors not find the fronto-striatal pathway? We think there might be two reasons. First, the thalamus and striatum play different roles in the development of reading ability. Previous meta-analyses have found that the thalamus is more involved in childhood reading processing, while the striatum is more involved in adult reading processing (Koyama et al., 2011; Hancock et al., 2017). The study of illiteracy training belongs to the early stage of reading development, so the thalamus is the most prominent and easy to discover. For example, another study of training based on functional illiteracy found that although the connectivity strength of the fronto-striatal and thalamo-visual pathways was significantly lower in the illiterate group than in the control group, only the thalamic network, not the striatum network, was enhanced after a short training session (Mohammadi et al., 2020). This finding suggests that the thalamic network is more likely to be increased early in training, while the striatum network requires more training time to become more involved in reading ability. This suggestion has also been confirmed in this study. Second, the selection of measuring characteristics may also be an important reason. The thalamus is an important relay station for sensory and perceptual processing, and nerve fibers project out of the thalamus to the cerebral cortex in all directions, allowing hub-like exchanges of information. Skeide et al. (2017) used a measure called degree to examine brain changes in illiterate individuals before and after training. Degree refers to the strength of connection between a brain region and other brain regions of the whole brain and is an important feature to measure hub attributes. As a result, variations in the thalamus are easier to identify. In this study, we used the striatum as the seed point for functional connectivity analysis and found variations in the fronto-striatal circuit associated with reading development. Similar findings were made by Alcauter et al. (2017) using a combination of independent component analysis and functional connectivity.

### Limitations

Several limitations of this study should be noted and need further research. First, in this study, we used a CLPM to examine the longitudinal relationship between attention and reading development after one year, which offered important empirical evidence for our understanding of the connection between attention and reading development. Future studies may further address this important question over a longer period. Second, the thalamus and striatum are relatively large subcortical structures and contain more subareas than other regions. In this study, we selected highly representative coordinates to characterize the thalamus and striatum based on previous studies and found thalamo-occipital and fronto-striatal circuits entailed two pathways for reading development. Future studies can fully explore all subareas of the thalamus and striatum to discover other possible pathways. Third, we found that reading could longitudinally predict the thalamus-occipital functional connectivity, but the thalamus-occipital pathways could not support or restrict reading development, which may be related to our sample being composed of normal developing children. Future research can adopt children with visual or reading difficulty to further verify whether the thalamo-occipital pathway could predict the longitudinal development of reading. Finally, the thalamo-occipital and fronto-striatal circuits are shared by reading and domain-general cognitive skills, but are not specific to reading development. Future studies should use caution when citing this conclusion.

## Conclusion

This study clarifies the vague description of the cortico-subcortical crosstalk that learning to read promotes and clearly describes two important pathways: one is the thalamo-occipital visual pathway centered in the thalamus, and the other is the frontal lobe-striatum cognitive pathway centered in the striatum. The former plays an important role in the early stage of learning to read and gradually decreases as reading ability improves, while the latter plays an important role in learning to read and gradually increases as reading ability improves.

## Data availability statement

The original contributions presented in this study are included in the article/supplementary material, further inquiries can be directed to the corresponding author.

## Ethics statement

The studies involving human participants were reviewed and approved by Institutional Review Boards at Beijing Normal University in accordance with the Declaration of Helsinki. Written informed consent to participate in this study was provided by the participants’ legal guardian/next of kin.

## Author contributions

ST, SPT, SQ, YH, and QD conceived and designed the study. YW, JL, LM, RC, and JW collected the data under the supervision of ST, SPT, WM, and J-HG. YW, JL, and LM performed data analysis under the supervision of CC and ST. YW, JL, LM, and ST wrote the manuscript. ST, YW, JL, LM, RC, and JW amend and proofread the draft of the manuscript. All authors reviewed and commented on the study and manuscript.

## Footnotes

1
http://rfmri.org/

## References

[B1] Alcauter S. Garcia-Mondragon L. Gracia-Tabuenca Z. Moreno M. B. Ortiz J. J. Barrios F. A. (2017). Resting state functional connectivity of the anterior striatum and prefrontal cortex predicts reading performance in school-age children. *Brain Lang.* 174 94–102. 10.1016/j.bandl.2017.07.007 28806599

[B2] Banaie Boroujeni K. (2021). *Mechanisms of cognitive flexibility in primate fronto-striatal circuits.* Nashville, TN: Vanderbilt University ProQuest Dissertations Publishing.

[B3] Binder J. R. Medler D. A. Westbury C. F. Liebenthal E. Buchanan L. (2006). Tuning of the human left fusiform gyrus to sublexical orthographic structure. *Neuroimage* 33 739–748. 10.1016/j.neuroimage.2006.06.053 16956773 PMC1634933

[B4] Bitan T. Cheon J. Lu D. Burman D. D. Gitelman D. R. Mesulam M. M. (2007). Developmental changes in activation and effective connectivity in phonological processing. *Neuroimage* 38 564–575. 10.1016/j.neuroimage.2007.07.048 17884585 PMC2638503

[B5] Bolger D. J. Perfetti C. A. Schneider W. (2005). Cross-cultural effect on the brain revisited: Universal structures plus writing system variation. *Hum. Brain Mapp.* 25 92–104. 10.1002/hbm.20124 15846818 PMC6871743

[B6] Brem S. Halder P. Bucher K. Summers P. Martin E. Brandeis D. (2009). Tuning of the visual word processing system: Distinct developmental ERP and fMRI effects. *Hum. Brain Mapp.* 30 1833–1844. 10.1002/hbm.20751 19288464 PMC6871060

[B7] Cupertino R. B. Soheili-Nezhad S. Grevet E. H. Bandeira C. E. Picon F. A. Tavares M. E. A. (2020). Reduced fronto-striatal volume in attention-deficit/hyperactivity disorder in two cohorts across the lifespan. *Neuroimage Clin.* 28:102403. 10.1016/j.nicl.2020.102403 32949876 PMC7502360

[B8] Dong Q. Lin C. D. (2011). *Standardized tests in children and adolescent mental development in China.* Beijing: Science Press.

[B9] Galandra C. Basso G. Manera M. Crespi C. Giorgi I. Vittadini G. (2019). Abnormal fronto-striatal intrinsic connectivity reflects executive dysfunction in alcohol use disorders. *Cortex* 115 27–42. 10.1016/j.cortex.2019.01.004 30738999

[B10] Gordon E. M. Laumann T. O. Marek S. Newbold D. J. Hampton J. M. Seider N. A. (2021). Human fronto-striatal connectivity is organized into discrete functional subnetworks. *bioRxiv* [Preprint]. 10.1101/2021.04.12.439415

[B11] Hamaker E. L. Kuiper R. M. Grasman R. P. (2015). A critique of the cross-lagged panel model. *Psychol. Methods* 20 102–116. 10.1037/a0038889 25822208

[B12] Hancock R. Richlan F. Hoeft F. (2017). Possible roles for fronto-striatal circuits in reading disorder. *Neurosci. Biobehav. Rev.* 72 243–260. 10.1016/j.neubiorev.2016.10.025 27826071 PMC5189679

[B13] Hidalgo-Lopez E. Pletzer B. (2021). Fronto-striatal changes along the menstrual cycle during working memory: Effect of sex hormones on activation and connectivity patterns. *Psychoneuroendocrinology* 125:105108. 10.1016/j.psyneuen.2020.105108 33516121

[B14] Houde O. Rossi S. Lubin A. Joliot M. (2010). Mapping numerical processing, reading, and executive functions in the developing brain: An fMRI meta-analysis of 52 studies including 842 children. *Dev. Sci.* 13 876–885. 10.1111/j.1467-7687.2009.00938.x 20977558

[B15] Koyama M. S. Di Martino A. Zuo X. N. Kelly C. Mennes M. Jutagir D. R. (2011). Resting-state functional connectivity indexes reading competence in children and adults. *J. Neurosci.* 31 8617–8624. 10.1523/JNEUROSCI.4865-10.2011 21653865 PMC3893355

[B16] Koyama M. S. Molfese P. J. Milham M. P. Mencl W. E. Pugh K. R. (2020). Thalamus is a common locus of reading, arithmetic, and IQ: Analysis of local intrinsic functional properties. *Brain Lang.* 209:104835. 10.1016/J.Bl.2020.104835 PMC8087146 32738503

[B17] Li G. Cheung R. T. Gao J. H. Lee T. M. Tan L. H. Fox P. T. (2006). Cognitive processing in Chinese literate and illiterate subjects: An fMRI study. *Hum. Brain Mapp.* 27 144–152. 10.1002/hbm.20173 16080160 PMC6871360

[B18] Li Y. Bi H. Y. (2022). Comparative research on neural dysfunction in children with dyslexia under different writing systems: A meta-analysis study. *Neurosci. Biobehav. Rev.* 137:104650. 10.1016/j.neubiorev.2022.104650 35367220

[B19] Mamiya P. C. Richards T. L. Edden R. A. E. Lee A. K. C. Stein M. A. Kuhl P. K. (2021). Task-related excitatory/inhibitory ratios in the fronto-striatal circuitry predict attention control deficits in attention-deficit/hyperactivity disorder. *medRxiv* [Preprint]. 10.1101/2021.03.25.21254355

[B20] Marcus D. S. Harms M. P. Snyder A. Z. Jenkinson M. Wilson J. A. Glasser M. F. (2013). Human connectome project informatics: Quality control, database services, and data visualization. *Neuroimage* 80 202–219. 10.1016/j.neuroimage.2013.05.077 23707591 PMC3845379

[B21] Martin A. Schurz M. Kronbichler M. Richlan F. (2015). Reading in the Brain of children and adults: A meta-analysis of 40 functional magnetic resonance imaging studies. *Hum. Brain Mapp.* 36 1963–1981. 10.1002/hbm.22749 25628041 PMC4950303

[B22] Mohammadi B. Munte T. F. Cole D. M. Sami A. Boltzmann M. Russeler J. (2020). Changed functional connectivity at rest in functional illiterates after extensive literacy training. *Neurol. Res. Pract.* 2:12. 10.1186/s42466-020-00058-0 33324918 PMC7650047

[B23] Muller-Axt C. Anwander A. von Kriegstein K. (2017). Altered structural connectivity of the left visual thalamus in developmental dyslexia. *Curr. Biol.* 27 3692–3698.e4. 10.1016/j.cub.2017.10.034 29153326

[B24] Ojha A. Parr A. C. Foran W. Calabro F. J. Luna B. (2022). Puberty contributes to adolescent development of fronto-striatal functional connectivity supporting inhibitory control. *bioRxiv* [Preprint]. 10.1101/2022.05.02.490303 PMC9730138 36495791

[B25] Power J. D. Barnes K. A. Snyder A. Z. Schlaggar B. L. Petersen S. E. (2012). Spurious but systematic correlations in functional connectivity MRI networks arise from subject motion. *Neuroimage* 59 2142–2154. 10.1016/j.neuroimage.2011.10.018 22019881 PMC3254728

[B26] Ren X. Z. Schweizer K. Xu F. (2013). The sources of the relationship between sustained attention and reasoning. *Intelligence* 41 51–58. 10.1016/j.intell.2012.10.006

[B27] Ren X. Z. Schweizer K. Wang T. F. Xu F. (2015). The prediction of students’ academic performance with fluid intelligence in giving special consideration to the contribution of learning. *Adv. Cogn. Psychol.* 11 97–105. 10.5709/acp-0175-z 26435760 PMC4591514

[B28] Richlan F. Kronbichler M. Wimmer H. (2011). Meta-analyzing brain dysfunctions in dyslexic children and adults. *Neuroimage* 56 1735–1742. 10.1016/j.neuroimage.2011.02.040 21338695

[B29] Rima S. Schmid M. C. (2020). V1-bypassing thalamo-cortical visual circuits in blindsight and developmental dyslexia. *Curr. Opin. Physiol.* 16 14–20. 10.1016/j.cophys.2020.05.001 PMC7617028 39649037

[B30] Rodrigue K. M. Daugherty A. M. Foster C. M. Kennedy K. M. (2020). Striatal iron content is linked to reduced fronto-striatal brain function under working memory load. *Neuroimage* 210:116544. 10.1016/j.neuroimage.2020.116544 31972284 PMC7054151

[B31] Siok W. T. Jia F. Liu C. Y. Perfetti C. A. Tan L. H. (2020). A lifespan fMRI study of neurodevelopment associated with reading Chinese. *Cereb. Cortex* 30 4140–4157. 10.1093/cercor/bhaa038 32108219 PMC7264688

[B32] Siok W. T. Niu Z. Jin Z. Perfetti C. A. Tan L. H. (2008). A structural-functional basis for dyslexia in the cortex of Chinese readers. *Proc. Natl. Acad. Sci. U.S.A.* 105 5561–5566. 10.1073/pnas.0801750105 18391194 PMC2291101

[B33] Siok W. T. Perfetti C. A. Jin Z. Tan L. H. (2004). Biological abnormality of impaired reading is constrained by culture. *Nature* 431 71–76. 10.1038/nature02865 15343334

[B34] Siok W. T. Spinks J. A. Jin Z. Tan L. H. (2009). Developmental dyslexia is characterized by the co-existence of visuospatial and phonological disorders in Chinese children. *Curr. Biol.* 19 R890–R892. 10.1016/j.cub.2009.08.014 19825347

[B35] Skeide M. A. Kumar U. Mishra R. K. Tripathi V. N. Guleria A. Singh J. P. (2017). Learning to read alters cortico-subcortical cross-talk in the visual system of illiterates. *Sci. Adv.* 3:e1602612. 10.1126/sciadv.1602612 28560333 PMC5443643

[B36] Tan L. H. Laird A. R. Li K. Fox P. T. (2005). Neuroanatomical correlates of phonological processing of Chinese characters and alphabetic words: A meta-analysis. *Hum. Brain Mapp.* 25 83–91. 10.1002/hbm.20134 15846817 PMC6871734

[B37] Tao S. (2019). Intelligence development and school adjustment of school-age children and adolescents: A follow-up cohort study. *Psychol. Commun.* 2 88–90.

[B38] Tao S. Liu H. Zhou C. Wang C. Sun C. Xu F. (2015). The roles of school psychological environment in grades 4∼ 6 students’ cognitive development: A multilevel analysis of the national representative data. *J. Psychol. Sci.* 1 2–10.

[B39] Tschentscher N. Ruisinger A. Blank H. Diaz B. von Kriegstein K. (2019). Reduced structural connectivity between left auditory thalamus and the motion-sensitive planum temporale in developmental dyslexia. *J. Neurosci.* 39 1720–1732. 10.1523/Jneurosci.1435-18.2018 30643025 PMC6391561

[B40] Vidyasagar T. R. (2019). Visual attention and neural oscillations in reading and dyslexia: Are they possible targets for remediation? *Neuropsychologia* 130 59–65. 10.1016/j.neuropsychologia.2019.02.009 30794841

[B41] Wang T. F. Ren X. Z. Schweizer K. Xu F. (2016). Schooling effects on intelligence development: Evidence based on national samples from urban and rural China. *Educ. Psychol.* 36 831–844. 10.1080/01443410.2015.1099618

[B42] Wang Y. Guan H. Ma L. Luo J. Chu C. Hu M. (2022). Learning to read may help promote attention by increasing the volume of the left middle frontal gyrus and enhancing its connectivity to the ventral attention network. *Cereb. Cortex* bhac206. 10.1093/cercor/bhac206 [Epub ahead of print]. 35641153

[B43] Xia C. H. Ma Z. Ciric R. Gu S. Betzel R. F. Kaczkurkin A. N. (2018). Linked dimensions of psychopathology and connectivity in functional brain networks. *Nat. Commun.* 9:3003. 10.1038/s41467-018-05317-y 30068943 PMC6070480

[B44] Xu J. Kemeny S. Park G. Frattali C. Braun A. (2005). Language in context: Emergent features of word, sentence, and narrative comprehension. *Neuroimage* 25 1002–1015. 10.1016/j.neuroimage.2004.12.013 15809000

[B45] Xu M. Wang T. Chen S. Fox P. T. Tan L. H. (2015). Effective connectivity of brain regions related to visual word recognition: An fMRI study of Chinese reading. *Hum. Brain Mapp.* 36 2580–2591. 10.1002/hbm.22792 25788100 PMC6869803

[B46] Yan C. G. Wang X. D. Zuo X. N. Zang Y. F. (2016). DPABI: Data processing & analysis for (Resting-State) brain imaging. *Neuroinformatics* 14 339–351. 10.1007/s12021-016-9299-4 27075850

[B47] Yan C. Zang Y. (2010). DPARSF: A MATLAB toolbox for “Pipeline”. Data Analysis of Resting-State fMRI. *Front. Syst. Neurosci.* 4:13. 10.3389/fnsys.2010.00013 20577591 PMC2889691

[B48] Zhang H. (2009). The revision of WISC-IV Chinese version. *J. Psychol. Sci.* 32 1177–1179. 10.16719/j.cnki.1671-6981.2009.05.026

